# Cash Transfers, Early Marriage, and Fertility in Malawi and Zambia

**DOI:** 10.1111/sifp.12073

**Published:** 2018-11-20

**Authors:** Fidelia Dake, Luisa Natali, Gustavo Angeles, Jacobus de Hoop, Sudhanshu Handa, Amber Peterman, on behalf of the Malawi Cash Transfer Evaluation Team and the Zambia Cash Transfer Evaluation Team

## Abstract

There is increasing interest in the ability of cash transfers to facilitate safe transitions to adulthood in low‐income settings; however, evidence from scaled‐up government programming demonstrating this potential is scarce. Using two experimental evaluations of unconditional cash transfers targeted to ultra‐poor and labor‐constrained households over approximately three years in Malawi and Zambia, we examine whether cash transfers delayed early marriage and pregnancy among youth aged 14 to 21 years at baseline. Although we find strong impacts on poverty and schooling, two main pathways hypothesized in the literature, we find limited impacts on safe transition outcomes for both males and females. In addition, despite hypotheses that social norms may constrain potential impacts of cash transfer programs, we show suggestive evidence that pre‐program variation in social norms across communities does not significantly affect program impact. We conclude with policy implications and suggestions for future research.

Diverse strategies are being employed globally to alleviate poverty and increase the well‐being of poor, vulnerable, and at‐risk populations. One such strategy that has gained popularity, particularly in the last decade, is providing cash transfers (both conditional and unconditional) to households, with programs reaching an estimated 718 million people in 2014 (World Bank 2015). Cash transfers help alleviate households’ short‐term needs, including food insecurity, acute poverty, and the educational needs of children, and facilitate the ability of households to make longer‐term investments such as productive investment in assets and small businesses, to reduce the intergenerational transfer of poverty (Baird, Ferreira, and Özler 2013; Bastagli et al. 2016; Davis et al. 2016; de Hoop, Groppo, and Handa 2017; Handa, Daidone, Peterman et al. 2018; Handa, Natali, Seidenfeld et al. 2018).

Although a large and robust literature shows positive impacts of cash transfers, debates regarding the existence and strength of domain‐specific impacts center around key design components. These include, for example, questions about optimal size and regularity of transfers, and complementary programming to boost effectiveness in relation to specific socioeconomic and contextual factors among beneficiary populations. In addition, there is increasing interest from the international community in the longer‐term impacts of cash transfers and their ability to facilitate safe transition to adulthood. This is particularly the case when focusing on females from poor households in low‐and middle‐income countries (LMICs) where early transitions, including into marriage and pregnancy, are an acknowledged problem (Salam et al. 2016). For example, an estimated 700 million women and girls globally were married before their 18th birthday, and an estimated 16 million adolescents give birth annually, 95 percent of which occurs in LMICs (UNICEF 2014; WHO 2014).

Impacts of cash support given to poor households on marriage and fertility among young people are plausible, both directly through decreases in poverty or indirectly through impacts on complementary domains, such as schooling (Baird, Ferreira, and Özler 2013; Rosenberg et al. 2015). The economic channel is the most obvious, as poverty has been shown to be both a cause and a consequence of early marriage and pregnancy among girls in LMICs. For example, a girl child may be regarded as an economic burden, thus marrying her off will relieve this financial burden (Birech 2013; Parsons et al. 2015). Additionally, in cultures where the payment of bride wealth is an integral part of the marriage process (as in many cultures in sub‐Saharan Africa), the income families receive may serve as an incentive for girls to be married off at younger ages during times of financial strain (Birech 2013). Further, education has been closely linked to early transitions, whereby completion of primary school has been found to delay marriage and fertility (Behrman, Parker, and Todd 2008), and early transitions may serve as a critical factor in deciding when to stop schooling (Lloyd and Mensch 2008).

The relative influence of economic and educational factors are, however, likely to vary by context, and these same pathways may be less important among male populations. In addition, it is often argued that one of the principal drivers of early transitions in sub‐Saharan Africa and in other LMICs, is normative cultural expectations around gender and family formation, rooted in historical social structures and reinforced by social norms (Birech 2013). However, while conforming to cultural expectations may provide benefits to youth and their families in terms of social standing, they are unlikely to outweigh the multitude of long‐term negative effects associated with early transitions (Wodon 2015).

Against the foregoing, we hypothesize that government‐run unconditional cash transfers have the potential to impact safe transitions among youth in sub‐Saharan Africa and we investigate this question by examining outcomes of early marriage/cohabitation and pregnancy among youth aged 14 to 21 in Malawi and Zambia. The programs in both countries were targeted to ultra‐poor, rural, labor‐constrained households and transferred cash on a bimonthly basis to heads of households. The evaluations were designed as cluster‐randomized controlled trials (cRCTs) and followed households over approximately three years from 2013 to 2015 in Malawi and 2011 to 2013 in Zambia. In addition to examining overall impacts, we further investigate if impacts differ by characteristics of youth or if cash affects main hypothesized pathways of poverty and education. Finally, in light of hypotheses that social norms may constrain potential impacts of cash programming, we examine program impacts by variation in social norms across communities.

Previous analysis examining the impacts of the Social Cash Transfer Program (SCTP) and Multiple Category Targeted Grant (MCTG) on primary outcomes has concluded that at the household level, both programs increased consumption, food security, material welfare and assets, and strengthened livelihoods and productive investment, while decreasing monetary poverty (UNC 2016; Fisher et al. 2017; Brugh et al. 2018; Handa et al. 2018). In addition, both programs were found to increase child school attendance, yet non‐negligible increases were also found in child work participation (both in household entrepreneurial activities and household chores) as children played a role in helping with household productive investment (de Hoop, Groppo, and Handa 2017; Kilburn et al. 2017). In addition, analysis from Malawi indicates that programs increased household resilience and ability to cope with shocks, and mental health of youth aged 13 to 19 at baseline (UNC 2016; Angeles et al. 2017). Despite these promising effects, a number of poverty‐related domains showed no effects resulting from the program, including nutritional status and health‐seeking behavior for under‐five children (AIR 2015; UNC 2016). Hence, there is evidence that programs in Malawi and Zambia were largely successful in meeting their main poverty‐related objectives. In addition, although several determinants of safe transitions have been analyzed utilizing these data (i.e., education and mental health as previously mentioned), primary outcomes related to reproductive health have not been examined in detail.

This article contributes to the literature in at least three ways. First, there is scarce evidence of at‐scale government‐run programs in sub‐Saharan Africa, and this analysis complements existing findings related to the SCTP and MCTG. To the best of our knowledge, the only other evidence to date from at‐scale government programs comes from Kenya's unconditional Cash Transfer for Orphans and Vulnerable Children (CT‐OVC) and from South Africa's Child Support Grant (CSG). The former utilizes cross‐sectional data at endline, while the latter uses a quasi‐experimental design. Therefore, our study is the first to use an experimental evaluation paired with longitudinal data to examine these questions from government programming in sub‐Saharan Africa. Second, we analyze data from both males and females—whereas the majority of the current literature focuses solely on females. Third, we explicitly estimate impacts on pathway indicators (poverty and education, to confirm that impacts from previous work are consistent within our sample) as well as potential program modifiers, including community‐level gendered social norms. To our knowledge, this is the first cash transfer evaluation examining outcomes of early marriage and pregnancy that explores and models the role of these factors.

## FRAMEWORK AND REVIEW OF THE LITERATURE LINKING CASH TRANSFERS, EARLY MARRIAGE, AND FERTILITY

**Figure 1:**
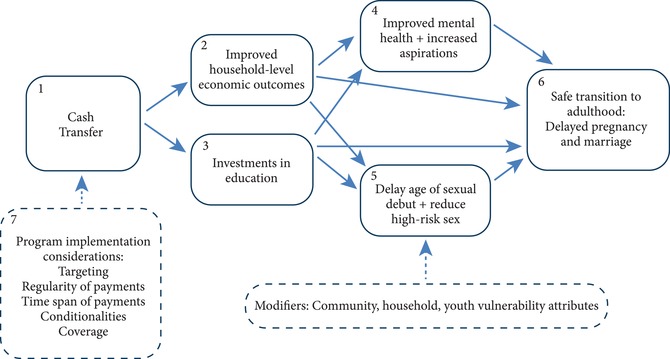
Conceptual framework for cash transfers and safe transitions to adulthood SOURCE: Adapted from Handa et al. 2015.

Frameworks connecting pathways or mechanisms between cash transfers and safe transitions (including early marriage and pregnancy) are generally broad and highlight two main proximate pathways: (1) poverty or economic security, and (2) investments in education. These two primary pathways are hypothesized to have the potential to directly reduce early pregnancy and marriage, as well as indirectly reduce the same, through secondary pathways of improved mental health and aspirations and delays in sexual debut and risky sex. We draw on a conceptual framework originally developed to understand linkages between the Kenyan Government's CT‐OVC and early pregnancy and marriage of female youth (Handa et al. 2015) to inform our analysis, as conceptually and operationally the programs examined here are similar. Figure 1 shows the pathways earlier described, linking cash transfers to safe transitions. As previously mentioned, there is a large and robust body of literature linking receipt of cash to both household‐level economic outcomes (Figure 1, Box 2) and human capital outcomes such as education (Figure 1, Box 3), across a wide range of program typologies and settings (Handa et al. 2015; Bastagli et al. 2016; Handa et al. 2016). Likewise, the relationship between economic security and safe transitions (Figure 1, Box 6), and increased education and safe transitions is fairly robust (Baird, Ferreira, and Özler 2013; Kilburn et al. 2016; Pettifor et al. 2016).

What is less well established is the relationship between cash transfers and improved mental and emotional health (Figure 1, Box 4), or risky sexual behaviour (Figure 1, Box 5), and linkages to transition outcomes. However, there is increasing evidence, primarily among female youth, that these links hold, particularly in the context of sub‐Saharan Africa (Baird et al. 2012; Baird, de Hoop, and Özler 2013; Handa et al. 2014; Kilburn et al. 2016; Pettifor et al. 2016). While a comprehensive review of pathways is beyond the scope of this article (and has been done elsewhere), we highlight several of the program design and contextual components relevant for this study (Handa et al. 2015).

One unique and important factor of the cash transfer programs in Malawi and Zambia is the targeting criteria for the beneficiary households. Because the programs target labor‐constrained households (i.e., households with a relatively high ratio of dependents to able‐bodied adults), they are likely to include a large number of adolescent and youth populations in addition to elderly members. These households are also often affected by the HIV epidemic, and many of the young people in these households are likely to be either orphans or have parents who are sick or nonresident (e.g., skipped‐generation households). This particular demographic may be vulnerable to early transitions including early pregnancy and marriage. The second important component to highlight is the potential role of modifiers, for example community, household, and youth‐specific vulnerability attributes. Here, we are particularly interested in the role of community‐gendered social norms around family formation, which may play a role in influencing the timing and nature of youth transitions—and therefore modify the potential impact of the cash transfer on these outcomes (Amin et al. 2017). In Malawi, Bosman (2011: 12) reports that early marriage can be viewed as “one of the customs embedded in the [country's] traditions and cultures,” and in Zambia, early marriage is socially acceptable if cultural and customary processes are followed (Mann, Quigley, and Fischer 2015). Further, girls in Malawi can be legally married at the age of 15 years with the consent of their parents, and since the payment of the bride price (*lobola*) provides a source of income in the immediate term, families prioritize early marriage of girls over education (Bosman 2011).

Two recent systematic reviews investigating “what works” to delay early marriage and prevent unintended and repeat pregnancy among young people in LMICs highlight few rigorous studies that are able to shed light on this question (Hindin et al. 2016; Kalamar, Lee‐Rife, and Hindin 2016). In total, 11 studies were found for interventions measuring early marriage in the published and gray literature (4 of which included cash transfers), and 21 studies were found for interventions measuring prevention of pregnancy (10 of which included cash transfers). Both systematic reviews conclude that cash transfers are promising instruments for facilitating safe transitions. However, they also acknowledge limitations, including a limited number of studies across program design typologies and geographic regions. Overall, the evidence from at‐scale cash transfer programs in sub‐Saharan Africa is particularly thin given the sheer number and coverage of government‐run large‐scale programs in the region (Cirillo and Tebaldi 2016).

Despite their potential, a unifying characteristic of large‐scale programming, particularly unconditional cash transfers, is that programs are typically designed around general poverty objectives and cash is given to the head of the household. More important, they do not have youth‐specific components, in contrast to the majority of NGO‐run programs focused on aspects of youth or girl empowerment, which may give transfers (including to girls themselves) and have additional features targeted to these objectives (Baird, McIntosh, and Özler 2011; Austrian et al. 2016; Pettifor et al. 2016). Thus, although current evidence points to a promising role for cash transfers in achieving impacts on early marriage and pregnancy in LMICs, in the case of poverty‐targeted at‐scale programs these should be seen as positive secondary impacts, in contrast to dedicated programs specifically aimed at achieving these objectives.

Building on recent reviews, we briefly summarize evidence from sub‐Saharan African countries, highlighting demonstrated effects of cash transfers on early marriage and pregnancy (Hindin et al. 2016; Kalamar, Lee‐Rife, and Hindin 2016). Most similar to the current study is an evaluation of the Government of Kenya's unconditional CT‐OVC, which examined transitions among 1,549 females aged 12 to 24 years after four years of the program (Handa et al. 2015). The study was a cRCT and found that females in treatment households were 5.5 percentage points (or approximately 34 percent) less likely to have ever been pregnant compared to their counterparts in control households. However, no significant treatment effects were found for early marriage or cohabitation. In Malawi, Baird and colleagues (2011) evaluated the impacts of the Zomba Cash Transfer Program, run by an NGO in the Zomba district of Malawi targeted at never‐married females aged 13 to 22 years. Using a cRCT with both an unconditional and a conditional treatment arm, the prevalence of marriage was found to reduce by 7.9 percentage points (44 percent) after two years in the unconditional treatment arm with no significant effects in the conditional arm. The study also observed that while about a quarter of females in the control group and the conditional arm were pregnant at the end of the evaluation period, among the unconditional treatment group, the likelihood of pregnancy significantly declined by 6.7 percentage points (27 percent; p < 0.01) (Baird, McIntosh, and Özler 2011). Pettifor and colleagues (2016) also used a cRCT and found that a cash transfer, conditional on schooling and targeted to young women aged 13 to 20 years enrolled in grades 8–11 at baseline in the Mpumalanga province of South Africa had no significant effect on incidence of pregnancy over the four‐year period of the study.

Other studies have, additionally, found promising results using quasi‐experimental methods. For example, Rosenberg and colleagues (2015) examined the relationship between South Africa's CSG and second pregnancy among recipients and nonrecipients using Cox regression models. Among the full cohort of 4,845 women, receipt of CSG was protective of second pregnancy in both the unadjusted and adjusted models (Hazard Ratios (HR) = 0.72 and 0.66, respectively) (Rosenberg et al. 2015). The protective effects of CSG receipt were also observed among women under 21 years in unadjusted and adjusted models (HR = 0.70 and 0.60, respectively). Another study conducted by Heinrich and colleagues (2017), using propensity score matching analysis revealed that receipt of CSG in adolescence reduced the probability of ever being pregnant by 10.5 percentage points among female adolescents (Heinrich, Hoddinott, and Samson 2017). In sum, there is promising evidence from the literature indicating that cash transfers have the potential to facilitate safe transitions, however the focus has been largely on females and there are few experimental studies from at‐scale government‐run programs.

## DATA AND METHODOLOGY

### Program and Evaluation Design

The main features of the two unconditional cash transfer programs and evaluations are summarized in Table 1. The SCTP in Malawi is the government's flagship social protection program and is targeted to ultra‐poor, labor‐constrained households. The main objectives are to reduce poverty and hunger, and to improve school enrollment rates. The program currently reaches over 330,000 households in all 28 districts in Malawi. The MCTG in Zambia is directed to households with a disabled member, or other vulnerable households such as those with a female or elderly head keeping orphans. The MCTG is implemented in two districts (Luwingu and Serenje) that have some of the highest food insecurity and poverty rates in the country. The primary goal of the MCTG is to reduce both extreme poverty and the intergenerational transfer of poverty. Both programs aim at helping the most vulnerable households meet their basic needs by providing regular bimonthly transfers that represent around 20 percent of baseline consumption. In Zambia, the transfer is a flat sum of 120 kwacha (ZMW) (US$24 per exchange rate at program start) per household bimonthly, whereas in Malawi, the transfer increases with household size (ranging from approximately 2,000 to 4,800 kwacha [Mk] with additional amounts for school‐age children) (US$5.80 to 13.30 per exchange rate at program start) bimonthly.

**Table 1:** Main features of cash transfer programs and evaluations

	Malawi	Zambia
Program	Social Cash Transfer Program (SCTP)	Multiple Categorical Targeted Grant (MCTG)
Implementer	Ministry of Gender, Children, Disability and Social Welfare	Ministry of Community Development and Social Services
Targeting	Ultra‐poor and labor‐constrained households^a^	Female‐ or elderly‐headed households keeping orphans; households with a disabled member; or special cases (critically vulnerable)
Transfer size	Variable by household size and number of children enrolled in school, bimonthly (∼18% of pre‐program consumption)^b^	Flat transfer of 120 kwacha [ZMW] (US$24), bimonthly (∼21% of pre‐program consumption)
Evaluation timeline	2013–2015 (30 months)	2011–2013 (36 months)
Location (stratification)	Two rural districts: Salima and Mangochi (two traditional authorities in each district)	Two rural districts: Luwingu and Serenje
Evaluation design	Cluster randomized‐controlled trial (cRCT); 29 villages (14 assigned to the treatment arm through public lottery)	Cluster randomized‐controlled trial (cRCT); 92 communities (46 assigned to the treatment arm through public lottery)
Household sample size	3,531	3,078

a Proxy means test (PMT) was used to target the ultra‐poor. A household is defined as labor constrained if it has a dependency ratio (ratio of “not fit for productive work” to “fit for productive work”) higher than three. “Unfit” household members are those below 19 years of age or above 64 years of age, or those between 19 and 64 years of age who have a chronic illness or disability, or are otherwise unable to work.

b After May 2015, the transfer size was adjusted up to 23% of average pre‐program consumption.

The evaluations of both programs were designed as cRCTs and carried out in two rural districts in each country covering 29 clusters in Malawi and 92 clusters in Zambia, of which approximately half were randomized via public lottery to a control group. A cluster is defined as a “community” in Zambia using the administrative unit of the Welfare Assistance Committee, and is defined as a “village cluster” in Malawi. The household sample size is 3,531 and 3,078 in Malawi and Zambia, respectively. Data were collected first at baseline, then at midline (after 17 months in Malawi and after 24 months in Zambia) and at endline (at 30 months in Malawi and at 36 months in Zambia). Due to delays in program implementation in Malawi, results can be interpreted as impacts after two years of program receipt. In Zambia, the impacts are after three years of program receipt. Further details regarding program and evaluation design can be found in technical reports accompanying the evaluations (UNC 2014; AIR 2012).

### Sample and Key Indicators

Both evaluations rely on multi‐topic household surveys and collected information regarding the socioeconomic status, health, and development outcomes of household members. Information on marital and pregnancy status comes from the household roster and fertility module, respectively (the latter of which collected data on all female household members aged 12 to 49). We focus on the panel youth—namely the youth for whom we have complete data both at baseline and endline. Sample sizes range from 878 and 1,023, respectively, for males and females in Malawi to 1,296 and 1,070 for males and females, respectively, in Zambia. These survey modules were typically administered to a well‐informed adult household member. For both males and females, we estimate the impact of the program on ever‐married or cohabited, defined as an indicator equal to 1 if the youth's reported marital status is married, co‐habiting, divorced/separated, or widowed, and 0 otherwise. For females, an indicator of ever being pregnant is equal to 1, if she is reported to have ever given birth, had a pregnancy that miscarried, was aborted, or ended in stillbirth, or if she is currently pregnant, and 0 if she has never experienced any of the previous. We further decompose the impact on ever being pregnant into the following three indicators: (1) ever given birth, (2) ever had a pregnancy that miscarried, was aborted, or ended in stillbirth, and (3) currently pregnant. We do not, however, show impacts on indicator (2) as it had very low means. We excluded inconsistent observations, namely those that at baseline report being “ever married” or “ever pregnant” but report contrasting information at endline.^1^

In addition to these outcome indicators, we examine several key pathway or moderating indicators including: (1) poverty, (2) education, and (3) community‐level gendered social norms. To measure poverty, we use household per capita consumption in logged local currency, which includes over 60 specific food and non‐food items (youth‐specific consumption was not collected). To measure education, we utilize standard indicators including whether the youth is in school (currently attending) and the highest grade attained. To measure community‐level gendered social norms, we created an index score through principal component analysis (PCA) using information on marriage arrangements (customary versus statutory) and inheritance rules for girls and women when their parents or husbands die^2^ (in reference to land, house, and other property from marriage and whether the widow can be inherited by the brother or other male relative of the deceased). The associated alpha values for the PCA are 0.81 (Malawi) and 0.84 (Zambia), indicating good performance of the index and correlation between individual indicators. Communities with scores above the mean are defined as “gender‐progressive communities.” Indicators used to build the community‐gendered social norm index vary between country, and descriptive statistics are reported at the cluster and individual level in Appendix B, Table B1.^3^ It is important to note that we did not register pre‐analysis plans for the analysis conducted here, pre‐specifying our modeling choices, and rather rely on robustness checks to ensure specific analysis choices do not unduly influence overall conclusions.

### Baseline Balance and Attrition

We focus on males and females aged 14 to 21 years for whom data was collected at baseline and then at follow‐up, after 30 and 36 months in Malawi and Zambia, respectively. This means that samples were approximately 16.4 to 16.9 years on average across countries at baseline and were approximately 19.4 to 19.8 years at follow‐up. We chose these age ranges as they represented the years where youth started to report marriage and pregnancy, however our main results are robust to including one or two additional year(s) more and less than the range for which results are shown. Table 2 (Panel A and B) shows baseline statistics for background characteristics for our analysis samples disaggregated by sex and country. Fifty‐two to 60 percent of the youth were attending school in Malawi compared to 58 to 68 percent in Zambia, with higher rates for males. In both programs, around 80 percent of the cash transfer recipients (and main respondents of the household questionnaires) are female and in their mid‐fifties with low formal education and high rates of out‐of‐marriage status (divorced, separated, or widowed). Household size is similar with approximately six members in both countries. Table 2 indicates that randomization was successful in creating balanced treatment and control groups at baseline. Across the 20 baseline background characteristics examined in Malawi and in Zambia (10 in each male and female panel), Wald tests indicate that only three are statistically significant at the p < 0.05 level (age, school attendance, and top half of community‐level gender progressiveness in the female panel in Zambia). The joint tests of orthogonality reported at the bottom of Table 2 confirm that pre‐program characteristics and primary outcomes are jointly uncorrelated with treatment assignment, with one exception, female youth in Zambia MCTG (p‐value < 0.01). Therefore, to account for any imbalance between groups and to improve the precision of estimates, we report adjusted estimates in all cases. Baseline reports further investigate baseline balance over a broad range of indicators (see AIR 2012 Appendix D, for Zambia; UNC 2014 Appendix F, for Malawi) and conclude that randomization was successful in creating two groups with similar characteristics.

**Table 2:** Baseline balance tests for key household characteristics and outcomes, by sex and country

	Male youth panel (14–21 years old at baseline)				Female youth panel^a^ (14–21 years old at baseline)			
	All	Control	Treatment	P‐value of diff.	All	Control	Treatment	P‐value of diff.
**Panel A: Malawi (Social Cash Transfer Program)**								
Youth: Ever married or cohabited (1/0)	0.02	0.02	0.02	0.93	0.12^b^	0.11^b^	0.12^b^	0.66^b^
Youth: Ever pregnant (1/0)	—	—	—	—	0.22	0.19	0.25	0.25
Youth: Currently pregnant (1/0)	—	—	—	—	0.04	0.03	0.06	0.09
Youth: Ever given birth (1/0)	—	—	—	—	0.18	0.16	0.20	0.30
Youth: Age (years)	16.52	16.50	16.55	0.71	16.38	16.44	16.32	0.43
Youth: Currently attending (1/0)	0.60	0.61	0.59	0.63	0.52	0.54	0.50	0.47
Main respondent: Age (years)	53.78	52.75	54.88	0.29	53.75	54.00	53.47	0.82
Main respondent: Female (1/0)	0.83	0.84	0.82	0.57	0.89	0.87	0.91	0.15
Main respondent: Ever attended school (1/0)	0.34	0.34	0.34	0.99	0.35	0.37	0.33	0.54
Main respondent, marital status: Divorced/separated/widowed (1/0)	0.62	0.60	0.64	0.55	0.61	0.6	0.62	0.75
Main respondent, marital status: Never married (1/0)	0.04	0.05	0.03	0.41	0.06	0.06	0.06	0.89
Household size	5.98	6.09	5.87	0.30	5.78	5.78	5.77	0.96
Highest (above mean) household monthly per capita consumption (1/0)	0.49	0.48	0.51	0.52	0.50	0.51	0.49	0.64
Gender‐progressive community (top half, 1/0)	0.49	0.52	0.46	0.77	0.49	0.51	0.47	0.83
Maganga (Traditional authority—Salima, 1/0)	0.24	0.28	0.20	0.64	0.24	0.28	0.19	0.62
Ndindi (Traditional authority—Salima, 1/0)	0.23	0.23	0.24	0.96	0.25	0.22	0.27	0.77
Jalasi (Traditional authority—Mangochi, 1/0)	0.29	0.25	0.34	0.68	0.27	0.23	0.31	0.66
Observations	1,023	549	474		917	492	425	
Joint orthogonality test (p‐value)	0.286	(0.988)			1.17	(0.393)		
**Panel B: Zambia (Multiple Category Targeted Grant)**								
Youth: Ever married or cohabited (1/0)	0.00	0.00	0.00	0.30	0.03^b^	0.03^b^	0.04^b^	0.22^b^
Youth: Ever pregnant (1/0)	—	—	—	—	0.09	0.09	0.10	0.42
Youth: Currently pregnant (1/0)	—	—	—	—	0.01	0.01	0.01	0.48
Youth: Ever given birth (1/0)	—	—	—	—	0.08	0.07	0.09	0.43
Youth: Age (years)	16.83	16.78	16.88	0.40	16.90	16.77	17.04	0.04
Youth: Currently attending (1/0)	0.68	0.68	0.68	0.98	0.58	0.61	0.53	0.02
Main respondent: Age (years)	54.81	54.73	54.91	0.90	54.04	54.40	53.63	0.65
Main respondent: Female (1/0)	0.76	0.76	0.76	0.97	0.78	0.78	0.79	0.88
Main respondent: Ever attended school (1/0)	0.71	0.72	0.69	0.48	0.68	0.67	0.68	0.78
Main respondent, marital status: Divorced/separated/widowed (1/0)	0.58	0.57	0.59	0.61	0.55	0.54	0.56	0.77
Main respondent, marital status: Never married (1/0)	0.03	0.03	0.03	0.99	0.04	0.05	0.03	0.33
Household size	6.29	6.43	6.14	0.25	6.26	6.27	6.25	0.96
Highest (above mean) household monthly per capita consumption (1/0)	0.53	0.55	0.52	0.46	0.53	0.54	0.52	0.63
Gender‐progressive community (top half, 1/0)	0.39	0.47	0.30	0.12	0.40	0.51	0.28	0.03
Serenje district (1/0)	0.52	0.54	0.50	0.70	0.56	0.57	0.55	0.81
Observations	1,296	663	633		1,210	630	580	
Joint orthogonality test (p‐value)	7.74	(0.085)			30.621	(0.006)		

NOTES: P‐values are reported from Wald tests on the equality of means of Treatment and Control for each variable. Standard errors are clustered at the community level. Sampling weights have been applied in Malawi. (1/0) depicts binary variables.

a We refer here to the female youth panel for early pregnancy, which is slightly larger than the panel for early marriage outcomes (878 in Malawi and 1,070 in Zambia).

b These estimates refer to the female youth panel for early marriage (878 in Malawi and 1,070 in Zambia), which is slightly smaller than the panel for early pregnancy outcomes used in this table.

— = Not applicable.

In addition to baseline balance, attrition poses a threat to internal validity and may cause bias in the estimation results. Attrition could be particularly important, given the age range of our sample, as youth are known to be a mobile population, and as our sampling protocol for the studies aims to follow households, not individual youth over time. In terms of household‐level attrition, 6.5 and 3.0 percent of the baseline sample was lost to follow‐up by endline in Malawi and Zambia, respectively. Therefore, overall household attrition is fairly low and evaluation reports investigate both household overall and differential attrition and conclude that neither is a concern (AIR 2015; UNC 2016). Table A2 in Appendix A indicates that overall attrition among youth in our analyses samples range from 19 to 29 percent depending on the sample and program (see Panel A). In Malawi, the proportion of youth that are lost to follow‐up in the treatment arm is similar to that in the control arm. However, in Zambia, there are more youth in the treatment group that were lost to follow‐up compared to their peers in the control group for both males and females.

These differential rates of attrition could be problematic if we believe that there are important reasons behind these differences that could be linked to our transition outcomes of interest. This is a plausible hypothesis, as both marriage and fertility could be a reason that youth leave households. For example, marriage in rural Zambia and Malawi is traditionally patrilocal (e.g., women move to live with or nearby the husbands’ family), however moves are often local or to neighboring areas (Cherchye et al. 2016). Furthermore, in Central and Southern Malawi (which makes up one study site in our sample, Salima district), matrilineal tribes dominate, where the opposite is true and men move nearby the wives’ family (Mwambene 2005). Therefore, we also investigated the reported reasons for youth leaving the sample over time, typically reported by the head of household (including the analytical reason of missing covariates or incomplete information, or the entire household moving, thus being dropped from the sample) and whether these differ between the treatment and control groups (Appendix Table A1). We report these results, and additional attrition analysis and robustness checks using inverse probability weights and construction of Lee bounds (Lee 2009) in Appendix A alongside discussion of results.

### Estimation Strategy

Building on the randomized experimental design of the intervention, we use an analysis of covariance (ANCOVA) specification to estimate the main impacts of the program on early marriage and fertility outcomes. When autocorrelation of outcomes over time is low, ANCOVA impact estimates are preferred over difference‐in‐difference estimates and provide a more efficient estimation of the effect (McKenzie 2012). In practice, autocorrelation in our samples for the outcomes of interest range from 0.37 (ever married male youth in Zambia) to 0.62 (ever married female youth in Zambia), and thus can be viewed as moderate. For this reason, we replicate our main findings using standard difference‐in‐difference models with household and individual fixed effects and find no differences in conclusions. The primary ANCOVA model is specified as follows:

(1) $$Y_{i,j,t_{1}}=\alpha +\beta T_{j}+\gamma Y_{i,j,t_{0}}+\sum_{k=1}^{K}\delta_{k}X_{k,i,t_{0}}+\theta_{s}+\varepsilon$$

where $Y_{i,j,t_{1}}$represents our outcome for the individual *i* in community or village *j* at follow‐up (*t*_1_); T is a dichotomous variable that takes the value 1 if the individual *i* lives in a community or village *j* assigned to receive the cash transfer and zero if the individual is assigned to a control community or village; β is therefore our coefficient of interest capturing the intent‐to‐treat (ITT) effect of the intervention. The ANCOVA specification controls for the pre‐treatment value of the outcome variable $Y_{i,j,t_{0}}$. $X_{i,t_{0}}$ represents the vector of control variables, all measured at baseline, that we included to increase the precision of our estimates and to account for pre‐program imbalances. Controls included in each program are listed in Table 2: the age and education (whether or not currently attending school) of the youth, characteristics of the main survey respondent or transfer recipient (age, sex, whether they have ever attended school, and marital status indicators), household size, a dummy capturing whether per capita consumption is below the mean, and an indicator that captures whether the youth lives in a gender‐progressive (top half of score) community. For fertility outcomes, we also included an indicator to capture whether the dependent variable (ever pregnant at endline, or subcomponents at endline) was missing and therefore replaced with midline data.^4^ However, replicating our main specifications without replacing outcome variables confirms our main results. Finally, $\theta_{s}$ captures strata fixed effects (traditional authorities and district indicators in Malawi and Zambia, respectively). We report adjusted and unadjusted specifications for our main analyses; unadjusted specifications maintain controls for the stratification indicators used for the randomization. We estimate using the ordinary least squares technique, Linear Probability Model (LPM) and adjusting standard errors for clustering at the cluster level. We use only baseline and endline data in our main ANCOVA models, therefore excluding the midline follow‐up, as we expect our outcomes to be fairly slow moving. However, we ran an alternative model where we pool the midline and endline and find the main results unchanged (not displayed).

To further investigate possible differential effects, we explore how levels of pre‐program poverty (“poorest” defined as those below mean consumption at baseline), education (currently attending school at baseline), and gender‐progressive norms at the community level affect impacts. In addition to these pathways, we also examine differences by pre‐program youth age, as adolescent outcomes are highly variable by age, with implications for targeting and timing of transitions. As we are interested in understanding whether the program had any differential impact across subgroups, we report the following specification:

(2) $$Y_{i,j,t_{1}}=\alpha +\beta T_{j}+\gamma Y_{i,j,t_{0}}+\sum_{k=1}^{K}\delta_{k}X_{k,i,t_{0}}+\vartheta C_{i,t_{0}}+\mu T_{j}C_{i,t_{0}}+\theta_{s}+\varepsilon$$

where $C_{i,t_{0}}$ represents a baseline characteristic of the youth (or the community where she/he lives), the coefficient μ on the interaction between the treatment status and the baseline characteristic captures whether the impact of the program varies between youth with a certain baseline characteristic and those without (for instance, it might capture the differential impact between the poorest half compared to least‐poor [above median] half).

## RESULTS

### Descriptive Results

The baseline values and balance for our key outcomes of interest are reported in Table 2, while Figure 2 is a graphic depiction of the mean outcomes by age, treatment status, and by country created using lowess regression (locally weighted scatterplot smoothing). The proportion of youth aged 14 to 21 years at baseline who have ever been married or pregnant is low at baseline on average (0 to 2 percent for early marriage among males; 3 to 12 percent for early marriage among females and 9 to 22 percent for early pregnancy). These averages are fully balanced at baseline between treatment and control groups (p > 0.05 level) and show increases with age, generally with a linear trend. Transitions appear to occur earlier within the Malawi sample across all outcomes and samples—for example, at baseline nearly 50 percent of the female sample is married or cohabiting at the age of 21 in Malawi, as compared to nearly 20 percent in Zambia. Likewise, at baseline, nearly 80 percent of the sample of girls aged 21 has ever been pregnant in Malawi, as compared to nearly 40 percent in Zambia. Although we cannot plot the confidence intervals (as lowess is based on an iterative smoothing procedure rather than on a single model), the proximity of the control (solid blue) and treatment (dashed red) lines suggest there is little difference for most outcomes with the treatment and control lines almost overlapping in both Zambia and Malawi at younger ages. There is larger dispersion among older ages with the control in Zambia showing less favorable outcomes (higher rates) for male early marriage and female pregnancy; however, standard errors are also larger at older ages due to the smaller samples.

Figure 2Early marriage and pregnancy outcomes over age by treatment status, at baseline and endline

**Figure:**
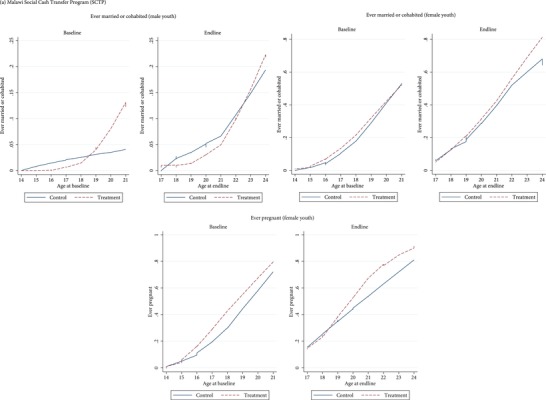

**Figure:**
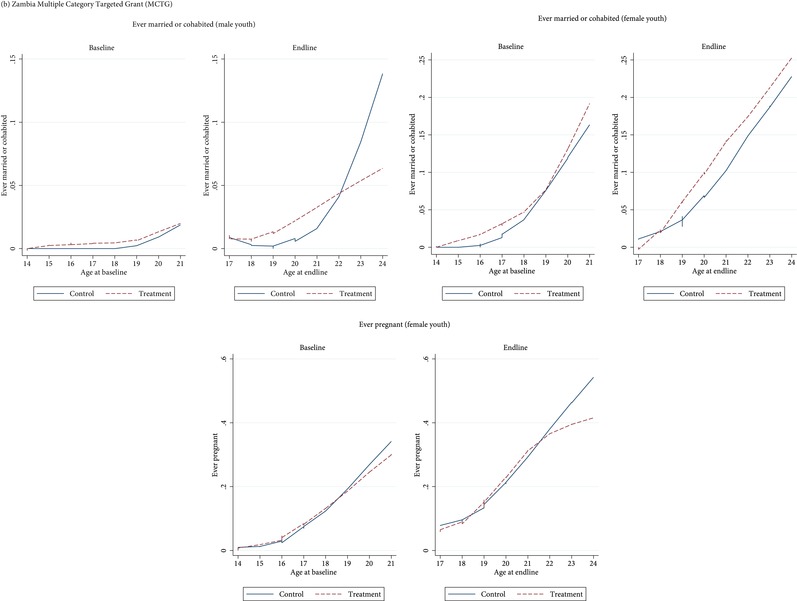

### Impact Estimates

Table 3 presents our main impact estimates for both unadjusted and adjusted models for Malawi (Panel A) and Zambia (Panel B). As suggested by the descriptive analysis, we find the cash transfer programs had no significant impact on early marriage and fertility outcomes in either country (see Table 3, Panel A and B). The only exception is a marginally significant negative impact on ever married or cohabited for male youth in the adjusted specification for Malawi. Full models by country are included in Appendix B (Tables B2 and B3). Furthermore, Tables B4 and B5 in Appendix B show robustness of our main findings to using a difference‐in‐difference model with individual and household fixed effects. We also examine robustness checks to account for potential differential attrition using inverse probability weights (see Appendix A, Tables A3 and A4) and Lee bounds (Appendix A, Table A5). We confirm the lack of significant impacts as presented in Table 3.

**Table 3:** Main impacts on early marriage and pregnancy among youth aged 14–21 at baseline, by sex and country

	Male		Female							
	Ever married or cohabited	Ever married or cohabited	Ever married or cohabited	Ever married or cohabited	Ever pregnant	Ever pregnant	Currently pregnant	Currently pregnant	Ever given birth	Ever given birth
	(1)	(2)	(3)	(4)	(5)	(6)	(7)	(8)	(9)	(10)
	Unadjusted	Adjusted	Unadjusted	Adjusted	Unadjusted	Adjusted	Unadjusted	Adjusted	Unadjusted	Adjusted
**Panel A: Malawi (Social Cash Transfer Program)**										
Treatment status	−0.0135	−0.0179^*^	−0.00983	−0.00428	−0.00137	0.00507	−0.0177	−0.0171	0.0196	0.0231
	(0.0111)	(0.00878)	(0.0187)	(0.0176)	(0.0238)	(0.0169)	(0.0174)	(0.0172)	(0.0234)	(0.0205)
Observations	1,023	1,023	878	878	917	917	917	917	917	917
R‐squared	0.371	0.403	0.364	0.454	0.403	0.463	0.094	0.100	0.379	0.447
Endline control mean	0.06		0.27		0.41		0.06		0.35	
**Panel B: Zambia (Multiple Category Targeted Grant)**										
Treatment status	0.000861	−0.00114	0.0112	0.0117	0.000860	0.000716	−0.0117	−0.0106	0.00375	0.00382
	(0.00779)	(0.00770)	(0.0145)	(0.0141)	(0.0187)	(0.0198)	(0.00957)	(0.0116)	(0.0174)	(0.0173)
Observations	1,296	1,296	1,070	1,070	1,210	1,210	1,210	1,210	1,210	1,210
R‐squared	0.140	0.167	0.398	0.415	0.362	0.398	0.117	0.133	0.386	0.424
Endline control mean	0.02		0.07		0.22		0.04		0.18	

NOTES: Estimations of equation (1) use ANCOVA modeling among panel individuals (follow‐up after 30 months in Malawi and after 36 months in Zambia). Robust standard errors in parentheses corrected for clustering. ^***^p < 0.01, ^**^p < 0.05, ^*^p < 0.1. All controls are measured at baseline and include stratifying indicators used for the randomization (traditional authorities dummies in Malawi and districts in Zambia). Inconsistent observations, namely those individuals reporting ever being married/pregnant at baseline but never being married/pregnant at endline were excluded from the analysis. Sampling weights have been applied in Malawi.

We further investigate whether the programs had any differential impact for subgroups of our sample. In particular, we distinguish youth by age (in years), between youth that lived in a gender‐progressive community at baseline and those who did not, between those who lived in a household with above mean consumption at baseline as well as youth who were attending school at baseline. Table 4 (Panel A and B) shows the interaction terms are not significant in either country or in any of the specifications reported. The only exception is the interaction between age and treatment (p < 0.1) in Malawi, indicating there may be a differential impact increasing the likelihood of ever being pregnant for older females with respect to the base category (younger females, who had not attended school, in low gender‐norm progressive communities). However, since the base coefficient is negative and larger than the interaction term—indicating an overall reduction—we cannot interpret this as an adverse effect.

**Table 4:** Heterogeneous impacts on early marriage and pregnancy, by country, education, and community gender‐progressiveness at baseline among youth aged 14–21 at baseline

	Panel A: Malawi (Social Cash Transfer Program)					Panel B: Zambia (Multiple Category Targeted Grant)				
	Male	Female				Male	Female			
	(1)	(2)	(3)	(4)	(5)	(6)	(7)	(8)	(9)	(10)
	Ever married or cohabited	Ever married or cohabited	Ever pregnant	Currently pregnant	Ever given birth	Ever married or cohabited	Ever married or cohabited	Ever pregnant	Currently pregnant	Ever given birth
Treatment status	0.0288	−0.315	−0.422*	−0.0179	−0.301	0.0790	−0.0301	0.0710	−0.0678	0.196
	(0.0863)	(0.221)	(0.234)	(0.140)	(0.241)	(0.0835)	(0.111)	(0.144)	(0.0794)	(0.137)
Youth: Currently attending (1/0)	−0.0366**	−0.201***	−0.209***	−0.0179	−0.179***	−0.0147	−0.0164	−0.0820**	−0.0353**	−0.0648*
	(0.0139)	(0.0501)	(0.0502)	(0.0265)	(0.0507)	(0.0148)	(0.0203)	(0.0342)	(0.0164)	(0.0346)
Youth: Currently attending (1/0)*Treatment status	0.00380	0.0405	0.0414	0.0396	−0.0206	0.00762	−0.0202	−0.00957	0.0263	−0.0190
	(0.0220)	(0.0660)	(0.0564)	(0.0341)	(0.0616)	(0.0198)	(0.0338)	(0.0440)	(0.0212)	(0.0413)
Youth: Age (years)	0.0140***	0.0272**	0.0256***	0.00261	0.0301***	0.0108**	0.0108**	0.0250***	−0.00204	0.0305***
	(0.00460)	(0.0102)	(0.00627)	(0.00532)	(0.00834)	(0.00421)	(0.00414)	(0.00612)	(0.00367)	(0.00577)
Youth: Age (years)*Treatment status	−0.00262	0.0207	0.0265*	−0.00151	0.0213	−0.00435	0.00437	−0.00360	0.00165	−0.00956
	(0.00586)	(0.0130)	(0.0132)	(0.00755)	(0.0128)	(0.00498)	(0.00641)	(0.00830)	(0.00426)	(0.00794)
Highest (above mean) household monthly per capita consumption (1/0)	0.0165	0.0533*	−0.00285	0.0137	−0.000235	−0.00212	0.00461	0.0147	−0.00682	0.0233
	(0.0216)	(0.0283)	(0.0280)	(0.0251)	(0.0279)	(0.0122)	(0.0163)	(0.0275)	(0.0155)	(0.0239)
Highest (above mean) household monthly per capita consumption (1/0)*Treatment status	−0.0255	−0.0689	−0.00125	0.0118	0.00468	−0.0192	−0.00831	−0.000747	0.0156	−0.0238
	(0.0251)	(0.0454)	(0.0413)	(0.0343)	(0.0374)	(0.0160)	(0.0256)	(0.0349)	(0.0208)	(0.0300)
Gender‐progressive community (1/0)	−0.0384***	−0.00760	−0.0355	−0.00727	−0.0339	−0.000149	0.0247	0.0412	−0.000634	0.0458
	(0.0117)	(0.0219)	(0.0298)	(0.0315)	(0.0315)	(0.00989)	(0.0197)	(0.0273)	(0.0143)	(0.0280)
Gender‐progressive community (1/0)*Treatment status	0.0144	−0.0327	−0.0587	−0.00161	−0.0361	−0.00513	−0.0452	−0.00962	0.0165	−0.0187
	(0.0177)	(0.0398)	(0.0437)	(0.0402)	(0.0564)	(0.0148)	(0.0323)	(0.0443)	(0.0255)	(0.0364)
Observations	1,023	878	917	917	917	1,296	1,070	1,210	1,210	1,210
R‐squared	0.404	0.458	0.466	0.102	0.450	0.169	0.417	0.398	0.135	0.424

NOTES: Estimations of equation (2) use ANCOVA modeling among panel individuals (follow‐up after 30 months in Malawi and after 36 month in Zambia). Robust standard errors in parentheses corrected for clustering. ^***^p < 0.01, ^**^p < 0.05, ^*^p < 0.1. All controls (and interactions) are measured at baseline and include stratifying indicators used for the randomization (traditional authorities dummies in Malawi and district dummies in Zambia). Estimations are adjusted and also include as controls the age and education of the youth, characteristics of the main survey respondent or transfer recipient (age, sex, whether they have ever attended school and marital status indicators), household size. Inconsistent observations, namely those individuals reporting ever being married/pregnant at baseline but never being married/pregnant at endline were excluded from the analysis. Sampling weights have been applied in Malawi.

Next we explore whether the program impacts reported in previous research holds within our sample, namely on poverty and education mediators. Using the same modeling and samples, we show in Appendix Table B6 that both programs have a large and significant positive effect on logged consumption per capita. In Zambia, the program increased consumption by 19 to 26 percent for youth in program beneficiary households compared to their control peers; in Malawi, the magnitude of the impact is even larger at around 30 to 31 percent. There is, therefore, concrete evidence that these poverty‐alleviation programs did indeed relax households’ liquidity constraints, thus making the mechanism of household‐level economic security feasible (UNC 2016; Handa et al. 2018). In addition, we estimate the program impacts on two education indicators in Appendix B, Table B7.^5^ In Malawi, cash transfers led to a positive impact on the proportion of male and female youth currently attending school in the range of 8 to 11 percentage points. A positive impact is also found in Zambia, however the impact is significant only for the male youth (for female youth at p < 0.1) and is smaller in magnitude, around 5 to 6 percentage points. We find no significant impacts on highest grade attained, however it is plausible that a longer time period is needed to see impacts on these longer‐term measures. Thus, we also find evidence that education is a plausible mechanism of program impact on safe transitions, albeit weaker evidence for long‐term measures of grade attainment (de Hoop et al. 2017).

## DISCUSSION AND CONCLUSION

We examined the impact of two government‐run unconditional cash transfer programs on early marriage/cohabitation and fertility among youth aged 14 to 21 in Malawi and Zambia. The programs share similar features, as they are targeted to extremely poor, labor‐constrained households in rural areas and provide bimonthly transfers to heads of households, with no additional program components or beneficiary responsibilities. After two and three years of transfers, there are few measurable impacts on safe transition outcomes for males or females, despite evidence of impacts on both household poverty and youth education outcomes, two of the most recognized mechanisms having the potential to drive impacts. Across countries and outcomes, the only significant impact found on the main outcomes of interest is a marginally significant protective impact on marriage or cohabitation for male youth in Malawi. Furthermore, despite hypotheses that suggest that transfers will be constrained by community‐level social norms relating to gender and family formation, we find no evidence suggesting this dynamic might be the case in our setting.

Our evidence is in contrast to the recent literature showing positive impacts of cash transfer programming included in recent reviews focused on early marriage and pregnancy in LMICs (Hindin et al. 2016; Kalamar et al. 2016). In fact, cash transfer programming can be viewed as one of the most promising interventions among those with evidence of sufficient rigor to be included. However, not all agree that cash transfer programming should be a preferred or favored strategy. For example, Amin and colleagues (2017: 11) argue that “while this approach [conditional cash transfers] has met with some success in improving education and health outcomes—it is unlikely, on its own to address the problem of child marriage.*”* Instead Amin and colleagues (2017: 11) suggest that a “multi‐dimensional, longer‐term and holistic view of impact*”* is needed to meet the Sustainable Development Goal (SDG) of ending child, early, and forced marriage by 2030. Despite the compelling call for multisectoral and girl‐focused programming, the primary focus and objective of large‐scale cash transfer programs such as the ones we evaluate is poverty reduction. Therefore, impacts on safe transitions should not be viewed as the marker of program effectiveness. To argue that (a narrow definition of) conditional cash transfer programs are alone unlikely to “eradicate” the problem of child marriage is misunderstanding both the objective of such programs, as well as disregarding the promise found in the larger evidence‐base showing marginal positive improvements across large‐scale populations. Even within girl‐focused programming, there is evidence that economic components are critical facilitators of positive impact. For example, a cRCT of a conditional financial incentive and girls’ empowerment curriculum in Bangladesh aimed at increasing girls’ education, and delaying marriage and childbearing, found that after 4.5 years of the program, girls receiving the financial incentive were 22 percent and 14 percent less likely to be married and have given birth, respectively—however, girls receiving the empowerment curriculum showed no meaningful changes on these same outcomes (Buchmann et al. 2016).

A number of explanations could account for our lack of significant findings. First, it is important to re‐emphasize that the sample is a unique population, within poor, rural districts of both countries and comprises youth who live in labor‐constrained households. For example, when compared to the most recent Demographic and Health Survey data, we find that rural females aged 17 to 24 (the same age as our cohort at endline) have prevalence of 68(60) percent for ever married and 72(76) percent for ever been pregnant in Malawi (Zambia) respectively (National Statistical Office [Malawi] and ICF 2017; Central Statistical Office [Zambia] et al. 2015). These figures are higher than represented in our sample, indicating that the youth in our sample, by virtue of being in labor‐constrained households, are not representative of the typical female demographic of reproductive ages. Further, as the study was designed to investigate dynamics primarily at the household level, individuals were not tracked over the study period. This means we are able to estimate impacts on early marriage and fertility only for the sample of youth who remain in study households. Although we perform a number of robustness checks and econometric tests to show that our sample is similar to youth who left, we rely on reports of household heads regarding their motivation for leaving. A stronger research design for these particular questions would track youth to investigate a fuller picture of these dynamics—and may in part have influenced the lack of significant findings in this analysis. Finally, as these outcomes are slow moving over time, it is possible that with a longer timeframe, additional transitions would give a different picture of dynamics and increase power to identify impacts. For example, it could be that cash transfers play a role in delaying transitions for several months up to a year, which could be argued as a meaningful change, however we would not necessarily pick up this marginal change with the current evaluation framework.

There are a number of additional limitations worth noting for future research. For example, we are not able to explicitly test additional pathways of mental health or sexual debut, or include subgroup analysis by orphan status, as these measures were not collected for our full sample of youth. In future work, it will be informative to test the full range of pathways and mechanisms responsible for impacts. In addition, the outcome measures are collected through self‐reports in household survey data and therefore are subject to social desirability and recall bias. Further, our measure of gendered social norms may not capture the full range of variability necessary to identify heterogeneous impacts with respect to our outcomes. We encourage future research to explicitly design measures to be able to more confidently address dynamics of social norms and their interactions with financial and economic security.

On a global level, there remain gaps in our understanding of effective programs and policy instruments to delay safe transitions, including early marriage and fertility in LMICs, particularly those that can be implemented at scale. Research suggests that poverty and economic interventions can play a meaningful role in facilitating safe transitions; however, evidence is limited in terms of relative costs and benefits of programs, program component synergies in the case of integrated or bundled programs, and few studies including men and boys. Despite the lack of impact demonstrated in Malawi and Zambia's national programs, this could be due to the unique study demographic, the length of time of the evaluation, or the study design, which allowed measurement of impacts only among youth who stayed in households. We welcome further rigorous research that has the ability to answer outstanding questions, to better understand how to improve outcomes for the next generation of young adults and their children.

## Supporting information

Supporting Information

## Footnotes

1 Excluded observations are limited in number. In Zambia, we dropped 10 observations in the male panel due to inconsistent reporting on our outcome variables; we dropped 38 and 54 in the female youth panel, for early marriage and early pregnancy outcomes, respectively. In Malawi, we dropped 10 observations in the male panel; 23 and 12 in the female youth panel, for early marriage and early pregnancy outcomes respectively.

2 In Zambia we use information regarding both daughters and wives, whereas in Malawi we rely only on information regarding widows, because information on daughters was not collected. We use baseline data for Malawi and 24‐month data for Zambia (on the assumption that gender norms are unlikely to change substantially over the two‐year time period).

3 Appendixes are available at the supporting information tab at wileyonlinelibrary.com/journal/sfp.

4 Endline missing values for “ever pregnant” were replaced with midline or baseline information in cases where the youth had already experienced a pregnancy. We created an indicator to capture whether the dependent variable had been replaced. For consistency across pregnancy subcomponents, whenever we replaced ever pregnant at endline, we also replaced the subcomponents.

5 Sample sizes in Table B7 are slightly smaller than those used in the main results table (Table 3). The main reason for this difference is that education questions at endline were asked only for household members aged 23 and younger; as a result, education outcomes are not available at endline for older youth.
