# Supplementary material for: Cash Transfers, Early Marriage, and Fertility in Malawi and Zambia
Source: Stud Fam Plann. 2018 Nov 20;49(4):295–317. doi: 10.1111/sifp.12073 (PMC6662603; doi:10.1111/sifp.12073)
Supplement: Supplementary file 1 — Supporting Information [file SIFP-49-295-s001.docx]

**APPENDIX A: Attrition**

In this Appendix, we report descriptive statistics on attrition (Table A1), further analysis on differential attrition (Table A2), and provide predicting equations and robustness checks using the inverse probability weighting (IPW) method to account for potential differential attrition (Tables A3 and A4). Finally, we construct upper and lower bounds for the intent-to-treat (ITT) effect following the Lee bounding approach (Table A5).

Table A1 shows that in Malawi, the main reported motivation for leaving the household for males is for work or school (58 percent), while for females it is marriage (50 percent). There are no statistically significant differences in terms of motivation for leaving the household among the male youth. However, in the female youth sample, we do observe a difference by treatment status: more female youth left the household for marriage reasons in the treatment group (56 percent in the treatment group versus 44 percent in the control group; p<0.04) and more female youth in the control group left the household for “other” reasons (6 percent in the control group versus 1 percent in the treatment group; p<0.01). In Zambia, the main reported motivation for leaving the households in the male youth sample is to live with other relatives (42 percent), while the main reason for females not being included in the study sample is due to missing or incomplete information (38 percent). Among all reasons for attrition, there is one statistically significant difference at the p<0.05 level: more households overall left the sample in the treatment sample (13 percent in the treatment group versus 5 percent in the control group; p<0.04).

We further explore individual attrition in Table A2. In Panel B, we examine differential attrition, or whether attrition is differentially correlated with baseline characteristics and outcomes by treatment status. Using baseline data, we model whether or not the individual is included in the panel using predictors of treatment status, pre-program background characteristics and primary outcomes (safe transition variables) as well as the interaction of treatment with each of these variables. The joint test of treatment and interaction terms in Panel B of Table A2 suggests no significant differential attrition for the female youth panels in Zambia, whereas in all panels in Malawi and in the male youth panel in Zambia, treatment seems to influence the characteristics of the individuals who remain in the panel. For this reason, we also examine robustness to attrition using IPWs.

Table A3 presents probit regressions of inclusion at follow-up for each sample (and outcome variable) in both countries. The dependent variable is a youth panel status dummy that equals 1 if the youth was surveyed both at baseline and follow-up and 0 if the youth was lost to follow-up (surveyed only at baseline). Covariates include the same set of baseline control variables included in our main specifications as well as other pre-program variables that are theoretically linked re-interview (“auxiliary variables”). Auxiliary variables include (1) the relationship of the youth to the eligible recipient of the cash transfer (if the youth is the biological child); (2) a proxy for the quality of the interview as measured by the non-self-clustered attrition rate; (3) covariate and idiosyncratic shocks (drought and flood measured at the community level, and death of the household income earner); and (4) the lagged dependent variable. For example, we hypothesize that if the youth is the biological child of the main cash transfer eligible adult in the household, he or she is more likely to remain in the household at follow-up. Likewise, if attrition rates are high in the overall cluster, a youth is more likely to also be lost to follow-up—either due to some unobserved community-level migratory trend, or because the particular survey team visiting the cluster is not as diligent at finding youth and completing call-backs for those found out of the household at the time of the survey visit.

The tests at the bottom of Table A3 indicate that auxiliary variables are jointly significant in explaining re-interview in each of the samples (specifications 1 to 6). For each specification, we then predict the probability of inclusion (Pi) and compute the IPW as the inverse of these predicted probabilities (Wooldridge 2002). These IPWs are then used as weights in the outcome regressions as a robustness check. Intuitively, IPWs correct for attrition by giving more weight to observations more likely to be underrepresented in the analysis panel. Thus, we would expect any bias due to differential attrition on observables to be corrected in the IPW impact estimates.

IPW results for each outcome, sample, and program are presented in Table A4. Findings using IPW are consistent with ITT estimates shown in the main text in Table 3; the lack of significant program impacts is confirmed, indicating that differential attrition is not driving our main results.

As a further robustness check to attrition, we construct upper and lower bounds for the ITT effect following Lee bounding approach (Lee 2009). In order to compute the bounds, we trim the distribution of our main outcome variables for the group (treatment or control) that is less likely to be lost to follow up by the relative difference in attrition rates between the two treatment arms. We trim the lower tail of the distribution to construct the upper bound and vice versa for the lower bound. Results are presented for each of our outcomes and program in Table A5 next to our main estimates. The majority of the bounds cross zero (7 out of 10). However, both lower and upper bounds are small and not statistically significant in most cases; whenever they are, Lee bounds are actually negative. We conclude that the main results of the article are broadly robust to attrition, with only few cases where bounds indicate that cash transfers might have actually had a positive protective impact on safe transitions (e.g., reducing pregnancy and marriage in some cases). Thus the bounded results indicate that our conclusion in the main text showing no significant program effects could be seen as conservative. However, it is important to note that while we present bounded results, we cannot be certain that the monotonicity condition for selection into attrition holds within our sample—e.g., that those lost to follow up are exclusively less or more likely to have favorable transition outcomes as compared to those in our panel sample. Thus, the bounded results should be interpreted with caution.

**Table A1. Reasons for attrition among those lost to follow-up, by sex and country**

**Table A2. Attrition analysis: Prediction of panel inclusion using treatment status (Panel A), background characteristics, and interactions between background characteristics and treatment (Panel B)**

**Table A3. Probit predicting re-survey (inverse probability weights), by program and sample**

**Table A4. Main impacts on early marriage and pregnancy among youth aged 14–21 at baseline, by sex and country, using IPW (inverse probability weights)**

**Table A5. Robustness of main impacts to attrition using Lee bounds**

**APPENDIX B: Supplemental Tables**

**Table B1. Means of indicators used to construct the gender norms score**

| **Panel A: Malawi, SCTP (n=28)** | **Community level** |
| --- | --- |
| Marriage type: matrilineal and neolocal | 0.049 |
| Marriage type: matrilineal and matrilocal | 0.877 |
| Marriage type: matrilineal and patrilocal | 0.074 |
| Inheritance rules for wife: Wife can inherit their land | 0.667 |
| Inheritance rules for wife: Wife cannot inherit their land | 0.333 |
| Inheritance rules for wife: Wife can inherit their house | 0.765 |
| Inheritance rules for wife: Wife cannot inherit their house | 0.235 |
| Inheritance rules for wife: Wife can inherit their other property from marriage | 0.784 |
| Inheritance rules for wife: Wife cannot inherit their other property from marriage | 0.216 |
|  |  |
| **Cronbach alpha (9 items)** | **0.809** |
| **Panel B: Zambia, MCTG (n=90)** | **Community level** |
| Most common marriage type: Customary marriage (with dowry/lobola) | 0.652 |
| Most common marriage type: Statutory marriage | 0.033 |
| Most common marriage type: Church wedding | 0.185 |
| Most common marriage type: Others | 0.022 |
| Most common marriage type: Eloping | 0.065 |
| Most common marriage type: Traditional marriage | 0.033 |
| Most common marriage type: Missing | 0.011 |
| Inheritance rules for daughter: Wife can inherit their land | 0.859 |
| Inheritance rules for daughter: Wife cannot inherit their land | 0.141 |
| Inheritance rules for daughter: Wife can inherit their house | 0.859 |
| Inheritance rules for daughter: Wife cannot inherit their house | 0.141 |
| Inheritance rules for wife: Wife can inherit their land | 0.848 |
| Inheritance rules for wife: Wife cannot inherit their land | 0.152 |
| Inheritance rules for wife: Wife can inherit their house | 0.837 |
| Inheritance rules for wife: Wife cannot inherit their house | 0.163 |
| Inheritance rules for wife: Wife can inherit their other property from marriage | 0.978 |
| Inheritance rules for wife: Wife cannot inherit their other property from marriage | 0.022 |
| Inheritance rules for wife: Widow can be inherited by brother or other relative of the deceased husband | 0.478 |
| Inheritance rules for wife: Widow cannot be inherited by brother or other relative of the deceased husband | 0.511 |
| Inheritance rules for wife: Missing | 0.011 |
|  |  |
| **Cronbach alpha (20 items)** | **0.840** |

**Table B2. Main impacts on early marriage and pregnancy among youth aged 14–21 at baseline, by sex in Malawi [full model]**

**Table B3. Main impacts on early marriage and pregnancy among youth aged 14–21 at baseline, by sex in Zambia [full model]**

**Table B4. Impacts on early marriage and pregnancy (individual fixed-effects), youth aged 14–21 at baseline, by sex and country**

**Table B5. Impacts on early marriage and pregnancy (household fixed-effects), youth 14–21 at baseline, by sex and country**

**Table B6. Potential mechanisms: Impacts on consumption per capita (logged), youth 14–21 at baseline by sex and country**

**Table B7. Potential mechanisms: Impacts on education outcomes (currently attending, highest grade attained), youth 14–21 at baseline, by sex and country**
